# Expression of p-Akt in ovarian serous carcinoma and its association with proliferation and apoptosis

**DOI:** 10.3892/ol.2013.1641

**Published:** 2013-10-25

**Authors:** TIEYING SONG, LIWEN WANG, ZHONGFU MO, LIMEI MAO, XIAOJING MA, RUNLING NIU, KUNFENG GU, RUYU YAN, PENGYU MA, YAN QI, QINGFANG JIAO

**Affiliations:** 1 Department of Anesthesiology, The First Hospital of Shijiazhuang, Shijiazhuang, Hebei 050011, P.R. China; 2 Department of Gynecology, The Sixth Hospital of Shijiazhuang, Shijiazhuang, Hebei 050000, P.R. China; 3 Department of Neurosurgery, The First Hospital of Shijiazhuang, Shijiazhuang, Hebei 050011, P.R. China

**Keywords:** phosphatidylinositol-3-kinase/protein kinase B, cyclin D1, proliferation, apoptosis, ovarian serous carcinoma

## Abstract

The aim of the present study was to determine the expression of p-Akt in ovarian serous carcinoma (OSC) and its association with proliferation and apoptosis. Paraffin-embedded tissues of patients aged between 35 and 64 years old without history of radiotherapy, chemotherapy and hormone therapy prior to surgery were collected. In total, samples included 12 ovarian serous cystadenomas (OSAs), 18 ovarian serous borderline tumors (OS-BTs) and 46 OSCs. Of the 46 OSC samples, 16 were well-differentiated, 20 were moderately differentiated and 10 were poorly differentiated, while 22 developed lymphatic metastases and 24 were metastasis-free. An additional 10 paraffin-embedded normal ovarian tissues (NOTs) were used as controls. Streptavidin-peroxidase immunohistochemistry assays were used to investigate the expression of p-Akt and cyclin D1 in the collected samples. Compared with NOT, p-Akt expression in the OS-BT and OSC groups, as well as cyclin D1 expression in the OSA and OSC groups, was significantly elevated (P<0.05). Compared with the OSA group, p-Akt expression in the OSC group was significantly elevated (P<0.01) and reversely associated with tumor differentiation (P<0.01), whereas cyclin D1 expression showed no correlation with tumor differentiation (P>0.05). The expression of p-Akt, caspase-3 and cyclin D1 was positively associated with lymphatic metastasis (r=0.334; P=0.023). The expression of p-Akt gradually increased with carcinoma development and was associated with differentiation and metastasis of OSC, revealing that the activation of the PI3K/Akt signaling pathway is involved in the development of OSC. Furthermore, the expression of cyclin D1 gradually increased in the NOT, OSA, OS-BT and OSC groups and was associated with tumor metastasis.

## Introduction

Ovarian carcinomas account for only 15–20% of female malignant carcinomas in the US; however, these tumors have the highest mortality rate. Ovarian serous carcinoma (OSC) accounts for 40% of all ovarian carcinomas in the US and is the leading cause of morbidity and mortality in malignant carcinoma of the female reproductive system (1). Despite applied chemotherapies and cytoreductive surgery combined therapies, the five-year survival rate of OSC is only 30–40% (2). Recent studies have found that ovarian carcinoma is a disease that is characterized by simultaneous excessive cell proliferation and decreased apoptosis, with uncontrolled proliferation and blocked apoptosis playing a crucial role in tumorigenesis (3,4). It is important to study the mechanisms and genes associated with proliferation and apoptosis in ovarian carcinoma, particularly for OSC, which may highlight new gene-therapeutic methods that simultaneously inhibit proliferation and induce apoptosis of cancer cells.

The phosphatidylinositol-3-kinase/protein kinase B (PI3K/Akt) signaling pathway was identified in 1987 by Staal who found an oncogene known as serine/threonine-specific protein kinase (Akt) in the murine retrovirus AKT8 after producing foci of malignant transformation in the mink lung epithelial cell line, CCL 64 (5). Akt is a 60-kDa serine/threonine-specific protein kinase prevalent in eukaryotic regulatory networks and has an important junction point which links multiple signal transduction pathways, regulates multiple extracellular cytokine signal transductions and is important for Ras-mediated oncogenic transformation (6). The PI3K/Akt signaling pathway is also involved in cell metabolism, regulation of the cell cycle and angiogenesis, and is associated with the development of diabetes and multiple autoimmune diseases, including rheumatoid arthritis. Deficiency or inactivation of the PI3K/Akt signaling pathway-associated regulatory genes, mutation or amplification of the PI3K gene and activation of receptors or junction molecules of its downstream signaling pathway have been identified in multiple tumor cell lines (7).

Cyclin-dependent kinases (CDKs) are crucial for the cell division cycle (8,9). The cell cycle is coregulated by CDKs and cyclin-dependent kinase inhibitors (CDKIs), as well as cyclin D1 levels, which are essential for the G1 to S phase cell cycle transition. However, little is known with regard to the association of Akt with the cell cycle progression.

Based on a previous study (10), pathomorphology and immunohistochemistry were used in the current study to investigate the expression of p-Akt and cyclin D1 in normal ovarian tissue (NOT), ovarian serous cystadenoma (OSA), ovarian serous borderline tumors (OS-BT) and OSC samples to further investigate the role of p-Akt in the development of ovarian epithelial cancer and its possible effect on cyclin D1 signaling pathway activation.

## Materials and methods

### Tissue samples

Paraffin-embedded tissues were collected from the First Hospital of Shijiazhuang (Shijiazhuang, China). All tissues originated from patients aged between 35 and 64 years old without history of radiotherapy, chemotherapy and hormone therapy prior to surgery. Among the collected samples, 12 were OSA, 18 were OS-BT and 46 were OSC. Of the 46 OSC samples, 16 were well-differentiated, 20 were moderately differentiated and 10 were poorly differentiated, while 22 OSC samples led to lymphatic metastasis and 24 were metastasis-free. An additional 10 paraffin-embedded NOTs were used as control. The current study was approved by the ethics committee of the First Hospital of Shijiazhuang and the Sixth Hospital of Shijiazhuang. Informed written consent was obtained from all participants.

### Immunohistochemistry

Immunohistochemistry S-P assays were used to investigate the expression of p-Akt in NOT, OSA, OS-BT and OSC samples. In total, 10 high power fields were randomly selected from each paraffin section and examined under a light microscope (Olympus, Tokyo, Japan) using the double-blind method. All slides were processed with polylysine prior to immunohistochemical staining for p-Akt and cyclin D1 protein visualization. Rabbit anti-human p-Akt monoclonal antibodies (Cell Signaling Technology, Inc., Danvers, MA, USA) and rabbit anti-human cyclin D1 monoclonal antibodies (ready-to-use; Fuzhou Maixin Biotechnology Development Co., Ltd., Fuzhou, China) were used as primary antibodies, and goat anti-rabbit IgG/biotin (Fuzhou Maixin Biotechnology Development Co., Ltd.) was used as the secondary antibody. Phosphate-buffered saline (PBS; 0.01 mol/l) only was used as a control for primary antibodies.

### Sample preparation

Paraffin-embedded samples were prepared into 4-μm sections and deparaffinized by a standard method. The paraffin sections were then submerged in hydrogen peroxide methanol solutions and vortexed at room temperature for 15 min to block the bioactivity of endogenous peroxidases. Next, sections were rinsed twice for 5 min with distilled water and placed into plastic boxes filled with antigen retrieval buffer (0.01 mol/l citric acid/sodium citrate solution, pH 6.0; Fuzhou Maixin Biotechnology Development Co., Ltd.) for initial microwave treatment at 7th gear for 5 min followed by a second treatment at 4th gear for 3 min. The boxes were then cooled at room temperature for 15–20 min. Next, sections were rinsed three times for 5 min with 0.01 mol/l PBS and normal goat serum was added at 37°C for 30 min to block endogenous biotin. Serum was discarded and primary antibodies (rabbit anti-human p-Akt monoclonal antibody, 1:200; and rabbit anti-human cyclin D1 monoclonal antibody, ready-to-use) were added separately for overnight incubation in a humid atmosphere at 4°C. Sections were then rinsed three times for 5 min with 0.01 mol/l PBS, and secondary antibody (goat anti-rabbit IgG/biotin) was added for 25 min at 37°C, followed rinsing three times for 5 min with 0.01 mol/l PBS. Next, streptavidin/horseradish peroxidase was added for 20 min at 37°C and the sections were rinsed four times for 5 min with 0.01 mol/l PBS. Finally, freshly prepared DAB-H_2_O_2_ (Fuzhou Maixin Biotechnology Development Co., Ltd.) was added for color development, which was monitored under a light microscope (Olympus CX21; Olympus Corporation, Tokyo, Japan) and rinsed again with distilled water to terminate the reaction. Hematoxylin (BASO Precision Optics Ltd., Taiching, Taiwan) was added for slight re-staining. Sections were differentiated with hydrochloric acid alcohol, dehydrated with an ascending series of ethanol, cleared in xylene and mounted in neutral balsam. Positive samples and negative controls were set up during the process. The procedure was used for all sample sections.

### Immunochemistry analysis

Selecting a homogeneously stained positive region and grading the proportion of stained cells compared with all cells within the field of vision was scored as follows: 0, no positively stained cells; 1, <25% positively stained cells; 2, 25–50% positively stained cells; and 3, >50% positively stained cells. Grading was determined by the color intensity of stained cells and was as follows: 0, negative; 1, weak light yellow; 2, medium brown yellow; and 3, strong dark brown. Results were analyzed by adding the above values together and were determined as follows: 0–2, negative (−); 3–4, weak-positive (+); and 5–6, strong-positive (++). Weak-positive (+) and strong-positive (++) were considered as positive.

### Statistical analysis

Data were analyzed using SPSS version 11.0 (SPSS, Inc., Chicago, IL, USA) and quantitative data are presented as the medium ± standard deviation. Values between groups were compared by one-way analysis of variance and counting data were analyzed by the χ^2^ test. P<0.05 was considered to indicate a statistically significant difference.

## Results

### Expression of p-Akt in ovarian carcinoma

**Figure 1:**
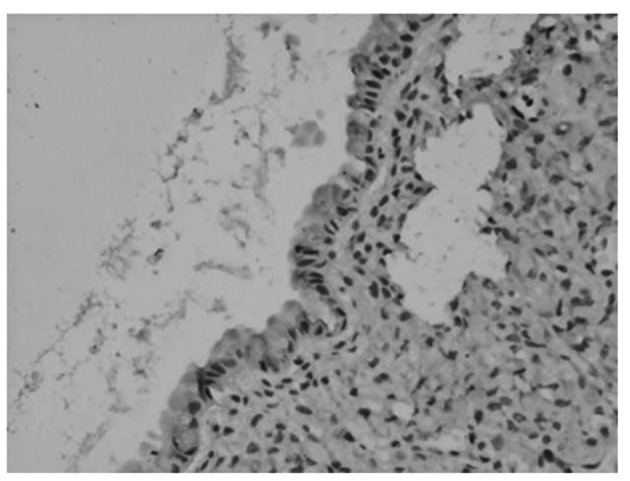
Negative expression of p-AKT in NOT (magnification, ×100). NOT, normal ovarian tissue.

**Figure 2:**
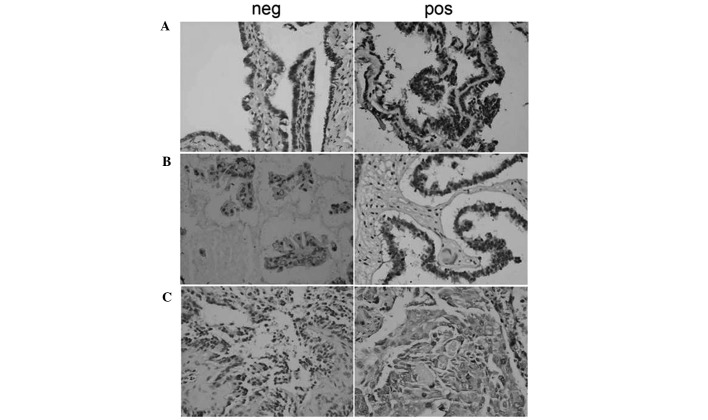
p-Akt expression patterns in (A) OSA, (B) OS-BT and (C) OSC. Left and right images represent negative and positive immunohistochemical staining, respectively (magnification, ×100). OSA, ovarian serous cystadenoma; OS-BT, ovarian serous borderline tumor; OSC, ovarian serous carcinoma.

p-Akt was predominantly located in the nuclei and cytoplasm of ovarian carcinoma cells, appearing as dark brown sediments (Figs. 1 and 2). Based on the immunohistochemistry S-P assay results (Tables I–III), p-Akt prevalence was significantly different between the NOT, OSA, OS-BT and OSC groups (χ^2^=19.781; P<0.01). In the OSC samples, the prevalence of p-Akt expression was reversely associated with tumor differentiation (P<0.01). p-Akt prevalence was positively associated with lymphatic metastasis (r=0.334; P=0.023) and a higher p-Akt prevalence was observed in OSC samples with lymphatic metastasis compared with metastasis-free OSC samples (P<0.05).

### Expression of cyclin D1 in ovarian carcinoma

**Figure 3:**
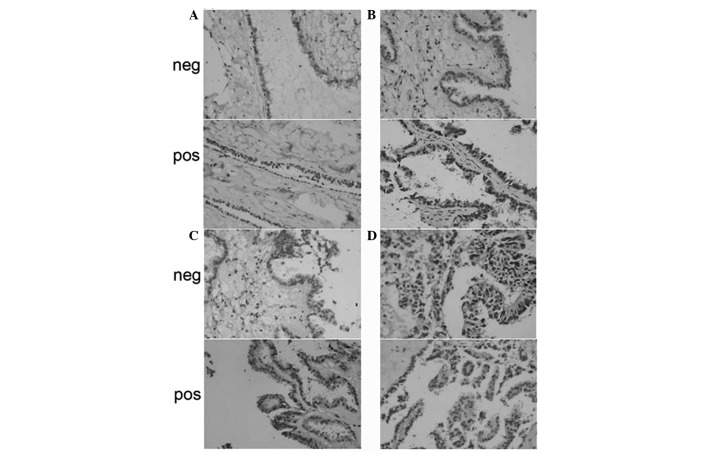
Cyclin D1 expression patterns in (A) NOT, (B) OSA (C) OS-BT and (D) OSC. Upper and lower panels represent negative and positive immunohistochemical staining, respectively (magnification, ×100). NOT, normal ovarian tissue; OSA, ovarian serous cystadenoma; OS-BT, ovarian serous borderline tumor; OSC, ovarian serous carcinoma.

Cyclin D1 was predominantly located in the nuclei and cytoplasm of ovarian carcinoma cells, appearing as brown nuclei and dark brown sediments in the cytoplasm (Fig. 3). Based on the immunohistochemistry S-P assay results (Tables IV–VI), the prevalence of cyclin D1 expression was significantly different among the NOT, OSA, OS-BT and OSC groups (χ^2^=19.241; P<0.01). OSA and OSC groups exhibited significantly higher cyclin D1 levels compared with the NOT group (P<0.05 and P<0.01, respectively). No significant difference in the prevalence of cyclin D1 expression was observed among the three tumor differentiation stages within the 46 OSC samples (P>0.05). The prevalence of cyclin D1 expression was positively associated with lymphatic metastasis (r=0.371; P=0.011), since a higher cyclin D1 prevalence was observed in OSC samples with lymphatic metastasis compared with metastasis-free OSC samples (P<0.05).

### Association of p-AKT, caspase-3 and cyclin D1

The prevalence of p-Akt expression was positively associated with the prevalence of cyclin D1 expression (P<0.001), but negatively associated with the prevalence of caspase-3 expression (P=0.017) (Tables VII and VIII).

## Discussion

The Akt gene, also known as protein kinase B, is an oncogene that was identified in 1987 by Staal and recognized as a serine/threonine-specific protein kinase (5). Akt was cloned in 1991 and is composed of an N-terminal regulatory domain, central kinase domain and C-terminal regulatory domain, as well as a hinge region. Phosphorylation of Akt at Ser473 and Thr308 is essential for the activation of p-Akt. Activated Akt relocates to the cytoplasm and nucleus where it phosphorylates multiple substrates to activate or inhibit downstream targets, including Bad (a Bcl-2 family member) (11), nuclear factor κB (12), glycogen synthase kinase-3 (GSK-3) (13), transcription regulatory proteins and other proteins involved in the regulation of cell proliferation, differentiation and apoptosis. Expression levels of p-Akt indicate the bioactivity of the PI3K/Akt signaling pathway. In the current study, immunohistochemistry S-P assays were used to detect the expression of p-Akt in ovarian carcinoma tissues. No p-Akt expression was identified in NOTs, but positive expression rates of 16.7, 55.6 and 82.6% were identified in OSA, OS-BT and OSC tissues, respectively. The positive expression rates of p-Akt in well-, moderately and poorly differentiated ovarian carcinoma were 43.6, 65.0 and 80.0%, respectively. The prevalence of p-Akt expression in ovarian carcinoma tissue with lymphatic metastasis was 81.8% and in metastasis-free ovarian carcinoma tissue was 50.0%. Statistical analyses indicated that the prevalence of p-Akt expression was significantly different among the NOT, OSA, OS-BT and OSC groups (χ^2^=19.781; P<0.01), particularly, between the NOT and OS-BT, OSA and OS-BT and OSA and OSC groups (P<0.01). Among the 46 OSC samples, p-Akt occurrence was negatively associated with the degree of tumor differentiation (P<0.01) and statistical analysis also revealed that p-Akt prevalence was positively associated with lymphatic metastasis (r=0.334; P=0.023), since OSC tissue with lymphatic metastasis exhibited significantly higher p-Akt levels compared with metastasis-free OSC tissue. Previous studies have reported a possible involvement of the PI3K/Akt signaling pathway in ovarian carcinoma development and its clinical implications. Philp *et al* found that the p85 subunit of PI3K may be a new ovarian carcinoma oncogene and that mutations in PI3KCA may play critical roles in the development of ovarian carcinoma (14). Based on immunohistochemistry assay results, Noske *et al* found that Akt expression was 58% higher in primary ovarian carcinoma compared with that in NOTs, and it was significantly associated with positive lymph node rates and International Federation of Gynecology and Obstetrics stages (15). In addition, western blot analyses revealed that positive Akt expression in all investigated ovarian carcinoma cell lines and gonadal hormones increased the invasion and metastasis of epithelial ovarian carcinoma cells via activation of the PI3K/Akt signaling pathway, which is consistent with the results of the current study.

Uncontrolled proliferation is a critical marker of malignant carcinoma, as vigorous proliferative activity of carcinoma cells is the basis and prerequisite of carcinoma invasion and metastasis. Proliferation of carcinoma cells is regulated by complex signaling pathways and dysfunctional cell cycle regulatory mechanisms. The cell cycle is coregulated by CDKs and CDKIs and a combination of cyclins with CDKs is essential for the activation of CDKs. Cyclin D1 is one of the most important cyclins, playing a crucial role in the transition in the G1/S cell cycle phase and controls the initiation of the cell cycle and mitosis completion (16,17). In the present study, immunohistochemistry S-P assays were used to detect the expression of cyclin D1 in ovarian carcinoma tissues and found an increasing trend in the positive staining rates of NOT, OSA, OS-BT and OSC samples from 10.0, 25.0, 55.6 to 73.9%, respectively. The positive rates of cyclin D1 in well-, moderately- and poorly-differentiated ovarian carcinoma were 68.8, 70.0 and 90.0%, respectively. Ovarian carcinoma tissue with lymphatic metastasis showed a 90.9% prevalence of cyclin D1 expression and a 50.0% prevalence in metastasis-free ovarian carcinoma tissue. Statistical analyses indicated that cyclin D1 expression was significantly different among the NOT, OSA, OS-BT and OSC groups (χ^2^=19.241; P<0.01). The prevalence of cyclin D1 expression in the OSA and OSC groups was significantly higher compared with that in the NOT group (P<0.05 and P<0.01, respectively). Among the 46 OSC samples, the prevalence of cyclin D1 expression did not significantly vary within the three tumor differentiation stages (P>0.05). An additional statistical analysis also revealed that cyclin D1 expression was positively associated with lymphatic metastasis (r=0.371; P=0.011), while cyclin D1 prevalence in OSC samples with lymphatic metastasis was significantly higher compared with metastasis-free OSC samples (P<0.05). Lee *et al* reported that the positive rates of cyclin D1 expression were increased in the NOT, OSA, OS-BT and OSC groups (P<0.05) and correlated with tumor differentiation, clinical stages and lymphatic metastasis, which is consistent with the results of the current study (18).

In the present study, the expression levels of p-Akt and cyclin D1 were analyzed in OSC samples and the prevalence of p-Akt expression was found to positively correlate with that of cyclin D1, indicating an association between the PI3K/Akt signaling pathway and OSC proliferation and apoptosis. In addition, activation of the PI3K/Akt signaling pathway in proliferation and apoptosis regulatory signal pathways was confirmed, which is consistent with previous studies. Akt directly regulates endogenous antiapoptotic effectors of the Bcl-2 family members and phosphorylates apoptosis cascade-related regulatory proteins that share the Bcl-2 homogenous domain 3. Bad belongs to this endogenous antiapoptotic Bcl-2 family, and p-Akt directly phosphorylates Bad by combining BH3 with apoptosis cascade-related regulatory proteins to further regulate protein bioactivity, inhibit antiapoptotic effects and induce apoptosis (19,20). Previous studies have indicated that p-Akt directly phosphorylates the prostate apoptosis response protein (Par-4) to inactivate apoptosis induction effects and maintain carcinoma cell survival. Forkhead box (Fox) proteins have conserved Akt phosphorylation sequences. Once Fox proteins are phosphorylated by Akt, they migrate out of the nucleus and chelate with cytoplasmic proteins, losing their facilitating effects on the transcription of apoptosis related genes, Fas-L and Bim, which induce apoptosis, arrest the cell cycle and stimulate metabolism (21). The PI3K/Akt signaling pathway blocks cyclin production or inhibits CDKI activity via multiple signaling pathways (22). p-Akt binds specifically to the p53 negative regulatory protein, MDM2, at Ser166 and Ser186 and relocates MDM2 to the nuclei. MDM2 interacts with p53, inducing its inactivation by blocking the arrest of p53 in cell cycle stage G1, thereby promoting the cell cycle. Akt phosphorylates GSK-3, which is continuously produced in resting cells, and induces the phosphorylation of cyclin D1 while being degraded by the endogenous proteasome. This results in an extended G1 stage, and p-Akt indirectly protects cyclin D1 by inactivating GSK-3. In addition, p-Akt enhances β-catenin stability, thereby improving the transcription efficiency of the LEF transcription factor, which results in the improved transcription and expression of cyclin D1 (23). To conclude, the present study indicates that the PI3K/Akt signaling pathway regulates the proliferation pathways of ovarian carcinoma cells, improving the proliferation activity and further enhancing invasion and metastasis. However, the PI3K/Akt signaling pathway also regulates the apoptosis-related proteins of carcinoma cells, which enhance the activation of endogenous antiapoptotic effectors and/or inhibit the expression and activation of apoptosis-associated proteases, thereby restraining apoptosis. The PI3K/Akt signaling pathway may play a key regulatory role in the development of OSC and become a primary target for gene therapy. Future in-depth studies may further contribute to the understanding of the mechanisms of ovarian carcinoma development and provide clinical guidance.

## Figures and Tables

**Table I:** Expression of p-Akt in different groups.

		p-Akt expression, n (%)		
			
Groups	n	−	+	P-value
NOT	10	10/10 (100.00)	0/10 (0.00)	
OSA	12	10/12 (83.33)	2/12 (16.67)	0.481
OS-BT	18	8/18 (44.44)	10/18 (55.56)	0.004
OSC	46	16/46 (34.78)	30/46 (65.22)	0.000

NOT, normal ovarian tissue; OSA, ovarian serous cystadenoma; OS-BT, ovarian serous borderline tumor; OSC, ovarian serous carcinoma.

**Table II:** Expression of p-Akt in different pathological grades of OSC.

		p-Akt expression, n (%)		
			
Pathological grade	n	−	+	P-value
G1	16	9/16 (56.25)	7/16 (43.75)	
G2	20	7/20 (35.00)	13/20 (65.00)	0.014
G3	10	0/10 (0.00)	10/10 (100.00)	

OSC, ovarian serous carcinoma.

**Table III:** Correlation between the expression of p-Akt and infiltration and metastasis of cancer tissue.

	p-Akt expression	
	
Infiltration or metastasis	−	+
−	12	12
+	4	18

r=0.334; P=0.023.

**Table IV:** Expression of cyclin D1 in different groups.

		Cyclin D1 expression, n (%)		
			
Group	n	−	+	P-value
NOT	10	9/10 (100.00)	1/10 (10.00)	
OSA	12	9/12 (75.00)	3/12 (25.00)	0.594
OS-BT	18	8/18 (44.44)	10/18 (55.56)	0.041
OSC	46	12/46 (26.09)	34/46 (73.91)	0.000

NOT, normal ovarian tissue; OSA, ovarian serous cystadenoma; OS-BT, ovarian serous borderline tumor; OSC, ovarian serous carcinoma.

**Table V:** Expression of cyclin D1 in different pathological grades of OSC.

		Cyclin D1 expression, n (%)		
			
Pathological grade	n	−	+	P-value
G1	16	5/16 (31.25)	11/16 (68.75)	
G2	20	6/20 (30.00)	14/20 (70.00)	0.161
G3	10	1/10 (10.00)	9/10 (90.00)	

OSC, ovarian serous carcinoma.

**Table VI:** Correlation between the expression of cyclin D1 and the infiltration and metastasis of OSC.

	Cyclin D1 expression	
	
Infiltration or metastasis	−	+
−	10	14
+	2	20

r=0.371; P=0.011. OSC, ovarian serous carcinoma.

**Table VII:** Correlation between the expression of p-Akt and cyclin D1 in OSC.

	Cyclin D1 expression	
	
p-Akt expression	+	−
+	28	2
−	6	10

r=0.606; P=0.000. OSC, ovarian serous carcinoma.

**Table VIII:** Correlation between the expression of p-Akt and caspase-3 in OSC.

	Caspase-3 expression	
	
p-Akt expression	+	−
+	8	22
−	6	10

r=−0.350; P=0.017. OSC, ovarian serous carcinoma.
